# New variants of alpha-1-antitrypsin: structural simulations and clinical expression

**DOI:** 10.1186/s12931-022-02271-8

**Published:** 2022-12-10

**Authors:** Angel Gonzalez, Irene Belmonte, Alexa Nuñez, Georgina Farago, Miriam Barrecheguren, Mònica Pons, Gerard Orriols, Pablo Gabriel-Medina, Francisco Rodríguez-Frías, Marc Miravitlles, Cristina Esquinas

**Affiliations:** 1 Department of Computational Medicine, Statistic Unit, Medicine Faculty, Universitat Autònoma de Barcelona, Bellaterra, Barcelona, Spain; 2 Department of Clinical Biochemistry, Hospital Universitari Vall d’Hebron, Vall d’Hebron Barcelona Hospital Campus, Barcelona, Spain; 3 Pneumology Department, Hospital Universitari Vall d’Hebron, Vall d’Hebron Institut de Recerca (VHIR), Vall d’Hebron Barcelona Hospital Campus, P. Vall d’Hebron 119-129, 08035 Barcelona, Spain; 4 Liver Unit, Department of Internal Medicine, Hospital Universitari Vall d’Hebron, Vall d’Hebron Institut de Recerca (VHIR), Vall d’Hebron Barcelona Hospital Campus, Barcelona, Spain; 5 Facultat de Medicina, Universitat Autònoma de Barcelona, Bellaterra, Barcelona, Spain; 6 Centro de Investigación Biomédica en Red de Enfermedades Hepáticas y Digestivas, (CIBEREHD), Barcelona, Spain; 7 Clinical Biochemistry Research Group/Vall d’Hebron Institut de Recerca (VHIR), Vall d’Hebron Barcelona Hospital Campus, Barcelona, Spain; 8 Centro de Investigación Biomédica en Red de Enfermedades Respiratorias (CIBERES), Barcelona, Spain; 9 Public Health, Mental, Maternal and Child Health Nursing Departament, Faculty of Medicine and Health Sciences, University of Barcelona, Barcelona, Spain

**Keywords:** Alpha-1 antitrypsin deficiency, SERPINA1 novel variants, Structural mapping, Molecular dynamic simulations

## Abstract

**Background:**

Alpha-1 antitrypsin deficiency (AATD) is characterized by reduced serum levels of the AAT protein and predisposes to liver and lung disease. The characterization at structural level of novel pathogenic SERPINA1 mutants coding for circulating AAT could provide novel insights into the mechanisms of AAT misfolding. The present study aimed to provide a practical framework for the identification and analysis of new AAT mutations, combining structural simulations and clinical data.

**Methods:**

We analysed a total of five mutations (four not previously described) in a total of six subjects presenting moderate to severe AATD: Gly95Alafs*18, Val210Glu, Asn247Ser, Pi*S + Asp341His and Pi*S + Leu383Phe + Lys394Ile. Clinical data, genotyping and phenotyping assays, structural mapping, and conformational characterization through molecular dynamic (MD) simulations were developed and combined.

**Results:**

Newly discovered AAT missense variants were localized both on the interaction surface and the hydrophobic core of the protein. Distribution of mutations across the structure revealed Val210Glu at the solvent exposed s4C strand and close to the “Gate” region. Asn247Ser was located on the accessible surface, which is important for glycan attachment. On the other hand, Asp341His, Leu383Phe were mapped close to the “breach” and “shutter” regions. MD analysis revealed the reshaping of local interactions around the investigated substitutions that have varying effects on AAT conformational flexibility, hydrophobic packing, and electronic surface properties. The most severe structural changes were observed in the double- and triple-mutant (Pi*S + Asp341His and Pi*S + Leu383Phe + Lys394Ile) molecular models. The two carriers presented impaired lung function.

**Conclusions:**

The results characterize five variants, four of them previously unknown, of the *SERPINA1* gene, which define new alleles contributing to the deficiency of AAT. Rare variants might be more frequent than expected, and therefore, in discordant cases, standardized screening of the S and Z alleles needs complementation with gene sequencing and structural approaches. The utility of computational modelling for providing supporting evidence of the pathogenicity of rare single nucleotide variations is discussed.

**Supplementary Information:**

The online version contains supplementary material available at 10.1186/s12931-022-02271-8.

## Introduction

A known genetic risk factor for chronic obstructive pulmonary disease (COPD) is severe alpha-1antitrypsin deficiency (AATD), a hereditary co-dominant autosomal disorder [1]. AATD is characterized by reduced serum levels of the alpha-1 antitrypsin (AAT) protein and predisposes to liver and lung disease [2]. AAT is synthesized at high levels by hepatocytes, and at lower concentrations by intestinal epithelial cells, neutrophils, lung epithelial cells and alveolar macrophages [3]. It is an important acute phase protein and the major serine protease inhibitor in human serum, in which it shows strong affinity to neutrophil elastase. The main site of AAT activity is in the lung, protecting the fragile connective tissue of the lower respiratory tract from the uncontrolled proteolysis triggered by neutrophils during inflammation. Importantly, AAT has also emerged as a multifunctional protein combining its inhibitory properties with immunomodulatory and anti-inflammatory activities [4].

AAT is encoded by the highly polymorphic *SERPINA1* gene, which is located on chromosome 14q32.1 and comprises four coding exons (II–V), three untranslated exons (Ia–Ic) and six introns [5]. The native AAT comprises 418 amino acids of which the initial 24 amino acids are a signal peptide and are subsequently removed. This results in a 394 amino acid mature protein of 52 kDa which is normally glycosylated at Asn46, Asn83 and Asn247 residues [6, 7]. The tertiary structure of AAT consists of 3 β-sheets (A–C), 9 α-helices (A–I) and a reactive central loop (RCL), which is an important region for initial interaction with the target protease [8]. This protein is characterized by a metastable native fold that is particularly sensitive to mutations [9, 10]. Single amino acid substitutions at specific positions of *SERPINA1* could favor the transition to inactive or polymeric dysfunctional folds linked to disease [11, 12].

Over 150 *SERPINA1* mutations have been identified to date, and of these, 60 are implicated in the pathogenesis of disease [13, 14]. The normal and non-disease-causing allele of AAT is called Pi*M and is present in more than 90% of the population. The most frequent deficient alleles are Pi*S (Glu264Val) and Pi*Z (Glu342Lys) [15, 16]. In particular, carriers of two Z alleles (Pi*ZZ) have strongly reduced circulating levels of AAT (up to 10–20% of normal, which is 1.3–2 g/L). Other rare alleles of AAT are also associated with reduced serum concentrations of the AAT protein, such as the Pi*Mmalton (Phe52del) and Pi*Siiyama (Ser53Phe), which also form intracellular polymers [17–19]. While the discovery of rare variants is continually increasing, especially in individuals with lower AAT concentrations, most remain of unknown clinical significance [20, 21].

In vitro characterization to define the pathogenicity of all AAT mutations identified is unlikely to be performed in some diagnostic laboratories [22]. However, computational modelling has shown to be a useful tool to characterize the potential deleterious effects of these not yet characterized variants and to provide a more precise diagnosis. In this regard, the combination of experimental and computational techniques has proven useful to study the influence of amino acid substitutions on the structure and function of mutated proteins through several examples [23]. Thus, the characterization at structural level of novel pathogenic *SERPINA1* mutations coding for circulating AAT could provide novel insight into the mechanisms of AAT misfolding in liver and lung disease, with important implications for molecular diagnosis and therapeutic development [24, 25].

The present study aimed to provide a practical framework for the identification and analysis of new AAT mutations in a clinical context, combining clinical data, genotyping and phenotyping assays, structural mapping and molecular dynamic (MD) simulations. Using this approach, we describe six novel uncharacterized AAT variants and delineate their potential role in the conformational dynamics of this protease inhibitor.

## Methods

### Subjects

All the patients were recruited from the outpatient clinic of the respiratory department of the Vall d’Hebron University Hospital (Barcelona, Spain) [26]. They were identified following the diagnostic algorithm for AATD used in our laboratory, which comprises three strategies: serum AAT quantification, protein phenotyping and genotyping [27].

Analysis by sequencing the entire encoding region of the *SERPINA1* gene is undertaken in patients with discrepancies between AAT concentration and phenotype pattern or allele-specific genotype. We identified six carriers of five variants (four not previously described): Gly95Alafs*18 in patient 1, Val210Glu in patients 2 and 3, Asn247Ser in patient 4, Pi*S + Asp341His in patient 5, and Pi*S + Leu383Phe + Lys394Ile in patient 6.

The study was approved by the Ethics Committee of the Vall d’Hebron Hospital (Barcelona, Spain) with the number PR(AG) 157/2016, and signed informed consent was obtained from all subjects for participation in the study.

### Clinical and molecular analysis

#### Clinical data and laboratory testing

Clinical characteristics and lung function data (forced expiratory volume in the 1st second (FEV1), forced vital capacity (FVC) and the FEV1/FVC ratio) were collected. Liver function was determined by analysis of liver enzymes: aspartate-aminotransferase (AST), alanine-aminotransferase (ALT), and gamma-glutamyl transferase (GGT). In addition, an enhanced liver fibrosis (ELF) test was performed. The ELF test (Siemens Healthcare Diagnostics, Vienna, Austria) is a fibrosis biomarker which consists of three components: type III procollagen peptide, hyaluronic acid, and tissue inhibitor of metalloproteinase-1 [28].

#### AAT serum concentrations and phenotyping

Quantitative measurement of AAT levels was determined by immune nephelometry, and phenotyping was performed with the isoelectric focusing (IEF) assay in serum samples in a pH gradient of 4.2–4.9 using the Hydragel 18 A1AT Isofocusing kit (Sebia). Comparison with sera of known phenotypes allowed determining whether the amino acid change affected AAT protein migration.

#### Genotyping of new alpha1-antitrypsin variants

For molecular studies, DNA was isolated from whole peripheral blood using a commercial extraction kit (Qiamp DNA blood, Qiagen). Sanger automated sequencing of all coding exons (II-V) of the *AAT* gene was performed as described previously [15]. New variants were identified comparing obtained sequences with the reference *SERPINA1* sequence (NG_008290.1, NM_000295.4).

#### Cloning of the new alleles

The genetic background of the Val210Glu, Pi*S + Asp341His and Pi*S + Leu383Phe + Lys394Ile was determined by cloning experiments. Polymerase chain reaction (PCR) products of exon II (Val210Glu) and exons III to V (Pi*S + Asp341His and Pi*S + Leu383Phe + Lys394Ile) from the respective patients’ DNA were cloned into a Zero Blunt TOPO vector, transformed into One Shot Top10 chemically competent *E*scherichia *coli* (Invitrogen) and sequenced by the usual protocol.

#### Circulating polymers of AAT

To quantify circulating polymers (CP), a sandwich ELISA has been performed using serum samples. 2C1 monoclonal antibody has been used to capture CP (HM2289 Alpha-1 Antitrypsin mAb 2C1), coating plates at 2 ug/ml overnight. The next day, the plates were incubated 2 h with 300ul/well with blocking solution. Standards and samples were diluted in blocking buffer, added to the plate, and incubated 2 h at room temperature. 3C11 monoclonal antibody labelled with horseradish peroxidase has been used to detect bound polymers (HM2358 Alpha-1 antitrypsin Human mAb3C11-HRP). After 80 min of incubation, 50ul/well of 3,3’,5,5’-tetramethylbenzidine (TMB) substrate solution were added (T0440, MERCK). AAT polymers (μg/mL) were determined by interpolation of absorbance values on the standard curve. Then, AAT polymers (%) were determined in all samples.

### Database search and computational modelling

#### Allele frequency

We investigated the presence and population frequency of the newly identified AAT variants in the Short Genetic Variations database (Single Nucleotide Polymorphism Database—dbSNP) (https://www.ncbi.nlm.nih.gov/snp/) and the Genome Aggregation Database (https://gnomad.broadinstitute.org/).

#### Structural modelling of AAT variants

Molecular models corresponding to missense AAT variants Val210Glu, Asn247Ser, Pi*S + Asp341His and Pi*S + Leu383Fhe + Lys394Ile were constructed with the Mutagenesis Wizard module of PyMOL 2.0.5 using the X-Ray crystal structures of the intact native wild-type human AAT at 1.8 Å and 2.0 Å resolution (PDBids: 1QLP, 3NE4) [29, 30]. Residues N-terminal to position 23 of the mature chain were disordered and lacked electron density in the template structures and were not modelled. Assignment of ionization states and hydrogens at physiological pH was conducted with the Protonate 3D application [31]. Molecular models were refined by energy minimization using the Amber12EHT forcefield to a gradient of 0.1 kcal/mol/Å within the Molecular Operating Environment (MOE) software suite (version 2020.09).

#### Molecular dynamic (MD) simulations

Molecular dynamics simulations in explicit solvent of wild type AAT and Val210Glu, Asn247Ser, Pi*S + Asp341His and Pi*S + Leu383Phe + Lys394Ile mutant structures were conducted with GROMACS [32]. Molecular systems were solvated with a simple-point charge water model in a rhombic dodecahedron box with monoatomic Na + and Cl− ions (0.2 M) and equilibrated with positional restraints on the protein backbone atoms using the Amber ff99SB-ILDN force field [33]. After relaxation, 1 microsecond of unbiased MD trajectories were produced at a constant temperature of 300 K and time step of 2 femtosecond. Analysis of MD trajectories was performed using GROMACS tools.

#### Electrostatic surface calculation

Electrostatic potentials of wild-type and mutant structures were obtained from the Adaptive Poisson-Boltzmann Solver (APBS) implemented in PyMol v2.5.0. (Schrödinger, LLC). Briefly, the APBS method uses the finite element method to numerically solve the nonlinear Poisson-Boltzmann equation, representing the electrostatic interactions between molecules in aqueous environments [34]. The solvent dielectric constant was 80, and the protein dielectric was approximated as 2.0. The isoelectric potential of − 5kT and 5kT was mapped onto the solvent accessible surface (1.4 Å sphere radii) of all the molecular models.

## Results

### Identification of new alpha1-antitrypsin variants

The entire coding sequence of the *SERPINA1* gene showed five novel variants (four not previously described) carried by six patients with AATD: four missense and one non-sense (Table 1). The clinical characteristics and the final genotype of the patients carrying the new variants are summarized in Table 2.

**Table 1:** Molecular description and final genotype of the new non well-described variants

Patient ID	Aa change in mature protein *	Aa change in precursor (NP_001121175.1)	Sequence position and change (NM_00025.5)	Exon	Genotype
Patient 1	Gly95fs	Gly119fs	355/356, GGC > GCT	II	G95Afs18* – M
Patient 2	Val210Glu	Val234Glu	701, GTG > GAG	III	V210E + H16H – M
Patient 3	Val210Glu	Val234Glu	701, GTG > GAG	III	V210E + H16H – M
Patient 4	Asn247Ser	Asn271Ser	812, AAT > AGT	III	N247S – M
Patient 5	S + Asp341His	S + Asp365His	1093, GAC > CAC	V	S + D341H – Z
Patient 6	S + Leu383Phe + Lys394Ile	S + Leu407Phe + Lys418Ile	1219, CTC > T TC 1253, AAA > ATA	V	S + L407F + K418I – M

*Aa* amino acid

^*^All variants are reported without the 24 amino acid corresponding to the signal peptide

**Table 2:** Clinical characteristics of patients carrying the new non well-described variants

Patient ID	Sex	Age	[AAT] (mg/dL)	AAT polymers (ug/ml)	AAT polymers%	Genotype	Smoking status	Pulmonary/hepatic status	Lung function (%)			Liver enzymes (IU/L)			ELF
									FVC	FEV1	FEV1/FVC	AST	ALT	GGT	
Patient 1	Female	49	78.1	3.65	0.47	G95Afs18* – M	Former smoker	– No evidence of liver disease	93	93	81	27	16	–	8.25
Patient 2	Male	40	76.3	2.52	0.30	V210E + H16H – M	Never smoker	– Mild hepatic steatosis – Elevated hepatic enzymes – No evidence of lung disease	93	101	87	72	108	98	8.67
Patient 3	Female	23	66.8	3.44	0.52	V210E + H16H – M	Never smoker	– Elevated hepatic enzymes – No evidence of lung disease	99	102	107	20	40	55	8.81
Patient 4	Male	58	98.4	5.20	0.53	N247S – M	Former smoker	– Mild hepatic steatosis and hepatomegaly – Normal hepatic enzymes – No evidence of lung disease	121	124	81	32	43	35	8.89
Patient 5	Male	45	19.8	4.08	2.06	S + D341H – Z	Former smoker	– Mild COPD, Bronchiectasis and recurrent pneumonia – No evidence of liver disease	98.6	81	62.9	16	18	17	7.62
Patient 6	Male	64	69.8	2.44	0,35	S + L407F + K418I – M	Never smoker	– Severe COPD Alcoholic liver disease	65.8	45.6	52	28	47	106	NA

*AAT* alpha-1 antitrypsin, *AST* aspartate-aminotransferase, *ALT* alanine-aminotransferase, *GGT* gamma-glutamyl transferase, *ELF* enhanced liver fibrosis, *NA* data not available, *FVC* forced vital capacity, *FEV1* forced expiratory volume in the first second, *COPD* chronic obstructive pulmonary disease

Normal ranges in serum: AAT: 116–2.00 mg/dL; AST: female 10 to 35 IU/L and male 10 to 50 IU/L; ALT: female 7 to 35 IU/L and male 8 to 50 IU/L; GGT: female 6–38 IU/L and male 9–55 IU/L; ELF score < 7.7: none to mild liver fibrosis, >  = 7.7 to < 9.8: moderate and >  = 9.8 sever

Patient 1 with the Gly95Alafs*18 variant in heterozygosity with the normal M allele was a 49-year-old female former smoker, with an accumulated exposure of 13 pack-years, and reduced AAT serum concentrations of 78.1 mg/dL (Table 1). She was referred from the respiratory unit showing normal lung and liver function.

Two unrelated patients carried the Val210Glu variant in heterozygosity. Patient 2 was 40-year-old non-smoker male. The liver unit carried out the AAT request due to mild hepatic steatosis showing elevated hepatic enzymes (AST: 72 U/L; ALT: 108 U/L; GGT: 98 U/L). The subject showed AAT serum levels of 76.3 mg/dL at the time of diagnosis and normal lung function after referral to the respiratory unit.

Patient 3 was a 23-year-old non-smoker female with a serum AAT concentration of 66.8 mg/dl. In this case, the digestive unit requested AAT quantification due to a likely liver disease and a mild hyper-transaminasemia (AST: 20 U/L; ALT: 40 U/L; GGT: 55 U/L). The patient showed normal lung function.

Patient 4 carried the Asn247Ser mutation and was a 58-year-old male former smoker of 10 pack-years with a serum AAT concentration of 98.4 mg/dL. He was referred from the digestive unit because of mild hepatic steatosis and hepatomegaly with normal liver enzymes and no evidence of lung disease.

Patient 5 carried the double mutation Pi*S + Asp341His and was a 45-year-old male former smoker with serum AAT levels of 19.8 mg/dL. He was initially diagnosed as a PI*ZZ phenotype at the age of 21. The patient suffered from bronchiectasis and recurrent pneumonias with mild airflow obstruction (FEV1/FVC: 63% and FEV1(%): 81% predicted). No signs of liver disease were detected. Due to the very low levels of AAT and the lack of genotyping at the time of diagnosis 24 years previously, the samples of the patient were tested again.

Patient 6 carrying the S + Leu383Phe + Lys394Ile variant was a 64-year-old male never smoker with AAT levels of 69.8 mg/dL. The individual showed alcoholic liver disease and the liver unit requested AATD analysis. The patient presented severe airflow obstruction (FEV1/FVC: 52% and FEV1 (%): 45.6% predicted).

### Protein migration phenotype

**Fig. 1:**
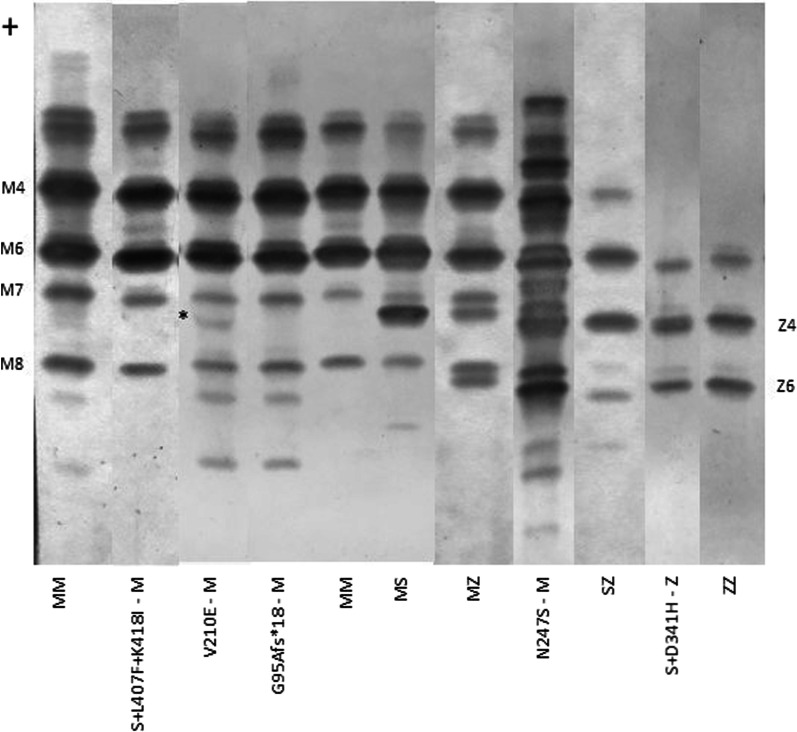
Isoelectric focusing electrophoresis of serum samples from subjects carrying the newly described variants and individuals carrying the most frequent normal and deficient genotypes. Serum from the V210E/M genotype showed an additional band (marked with asterisk) not present in the MM control. As expected, G95fs/M displayed a MM phenotype. Variants carrying a new mutation with S allele in cis configuration (S + L407F + K418I/M, S + D341H /Z) had null phenotype behavior, showing a MM and ZZ phenotype, respectively. The N247S/ genotype presented a multiband phenotype, different from the pattern in MM, MS, MZ, SZ or ZZ. Numbers at the right and left represent the migration of major (4 and 6) and minor (7 and 8) bands from the MM and ZZ genotypes

Figure 1 shows the phenotype determined by IEF assay in serum samples. Patient 1 carrying the null variant (Gly95Alafs*18) showed a PI*MM phenotype, corresponding to the PI*M variant carried by the patient. This variant seems to produce complete degradation of the truncated protein, a mechanism usually observed in null alleles.

Patient 2 with the Val210Glu variant displayed an IEF pattern similar to that of PI*MM. However, an extra band was observed between the M7 and M8 bands. The two bands after M8 that migrate more cathodally are often observed in the PI*M allele. Regrettably, a plasma sample from patient 3 carrying the Val210Glu variant was not available for IEF.

Patient 4 (Asn247Ser) showed a multiband pattern with bands associated with PI*M and PI*Z alleles, among others. This migration pattern may be explained by the alteration of protein glycosylation due to the omission of the N-glycosylation site at position 247 of the mature protein (see below).

Patient 5 carrying the Pi*S + Asp341His variant showed a PI*ZZ phenotype pattern, which could explain the initial diagnosis of this patient. The combination of the S allele with Asp341His seems to produce a null phenotype, in which only the PI*Z allele, in trans-configuration with the new variant, is observed.

Finally, Patient 6 (Pi*S + Leu383Phe + Lys394Ile) was phenotyped as PI*MM. Similar to patient 5, the combination of the S variant in cis-configuration with the new variants had features of a null phenotype behavior, in which only the M allele was observed.

### Genomic location and allele frequency

#### Patient 1; Gly95Alafs*18

Molecular study of the *SERPINA1* gene revealed a G base deletion in exon II at nucleotide position 355 or 356 (NM_000295.5, c.355/356delG), resulting in a frameshift that gives rise to a new stop codon downstream the deletion site at amino acid position 112, leading to a premature termination of protein translation. This variant occurred in heterozygosity with the presence of one normal M allele and has not been previously reported. The dbSNP entry rs749295615 indicates three different amino acid substitutions at this location.

#### Patients 2–3; Val210Glu

DNA sequencing revealed an A > T transversion in exon III (NM_000295.5, c.701 T > A) in heterozygous state with the M variant. This change produces a substitution of valine to glutamic acid (p.Val210Glu) in the mature protein. Moreover, both patients carried a synonymous mutation in exon II (c.120 T > C, p.His16His), and cloning experiments using a sample from patient 2 demonstrated that this change was in cis-configuration with the Val210Glu variant. This mutation was previously detected in heterozygous state with the Z variant in a 75-year-old male who had atelectasis and COPD with serum AAT levels of 42 mg/dL and was named M1pierre-bénite [35]. The Val210Glu change has been reported with a low frequency (< 1/10,000) in dbSNP (rs746197812).

#### Patient 4; Asn247Ser

This amino acid mutation is characterized by an A to G transition in exon III (NM_000295.5, c.812 A > G), resulting in an asparagine to serine substitution (p.Asn247Ser). This single nucleotide variation (SNV) was found in heterozygous form with M and has not been previously reported. However, the accession rs755851961 documented a change of asparagine to aspartic at the same position. Asn247 is one of the three N-glycosylation sites identified in AAT [6].

#### Patient 5; Pi*S + Asp341His

Gene sequencing demonstrated that this patient was a carrier of three alleles: PI*S, PI*Z, and the new variant. This new allele consisted of a G > C transversion in exon V (NM_000295.5, c.1093 G > C), changing aspartic acid for histidine at codon 341 (p.Asp341His) in the mature protein. Cloning experiments showed that this new variant was in cis-configuration with the S allele and in trans-configuration with the Z variant. The change Asp341His is found with a low frequency (< 1/10,000) in dbSNP (rs143370956). This position seems to be highly polymorphic as rs864622046 and rs201774333 indicate additional substitutions at this site.

#### Patient 6; Pi*S + Leu383Phe + Lys394Ile

Exon sequencing of the *SERPINA1* gene showed two single base substitutions at the end of exon V. This new allele consisted of a C > T transition at nucleotide position 1219 (NM_000295.5, c.1219 C > T) and an T > A transversion at position 1253 (NM_000295.5, c.1253 A > T) leading to leucine to phenylalanine (Leu383Phe) and lysine to isoleucine (Lys394Ile) changes in the mature protein, respectively. Background analysis confirmed that both mutations were in cis-configuration with the S variant and in trans-configuration with the M allele. Variant Leu383Phe was not found in genomic databases, but entries rs767700105 and rs1011476837 documented one synonymous and one missense (Leu383Arg) substitution at this position. On the other, hand, the mutation Lys394Ile is described with a low frequency (< 1/10,000) in dbSNP (rs1566747286).

### Circulating polymer concentrations in the different AATD genotypes

Serum samples from all patients were analyzed. Patients 4 and 5 presented higher CP concentrations. However, the concentration had to be normalized taking into account the AAT levels of each patient. Considering the percentage of total AAT, patient 5 (variant S + D341H – Z) presented the highest CP concentration (2.06%). The other variants presented between 0.3% and 0.5% of CP concentrations (Table 2).

### Structural mapping and molecular dynamic (MD) simulations

AAT is a conformationally flexible protein with several regions involved in a large structural rearrangement necessary for its protease inhibitory function. Therefore, this protein is susceptible to mutations that cause misfolding, inactivation or intracellular accumulation of pathogenic polymers [36–38].

**Fig. 2:**
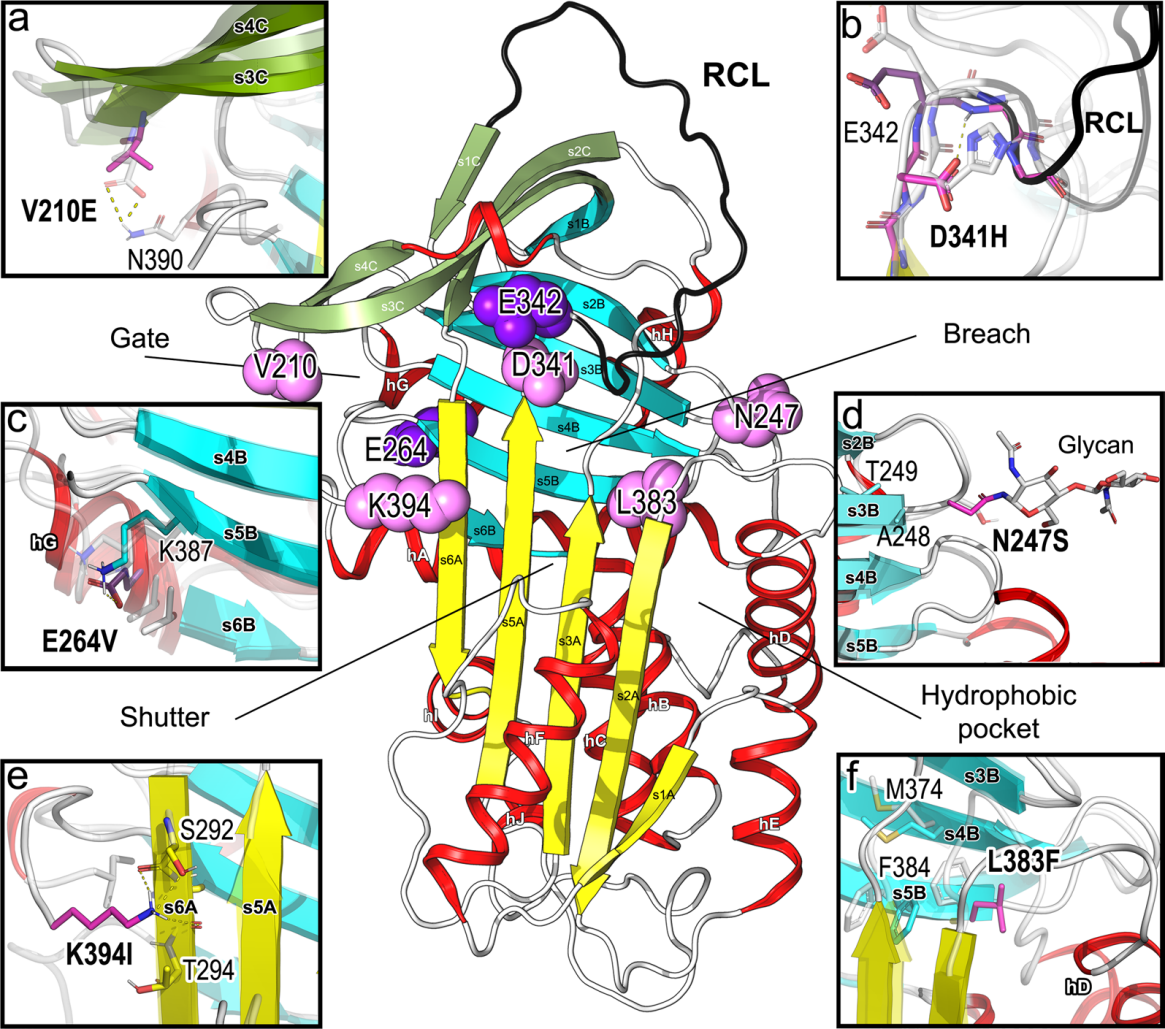
Structural mapping and analysis of the AAT variants identified. The central image is a cartoon representation of the molecular coordinates of human AAT1, with the serpin RCL shown in black, alpha-helices in red and beta-sheet regions A, B and C in yellow, cyan, and green, respectively. Mutated residues are highlighted in vdW surface (light violet = new variants; deep purple = Pi*Z and Pi*S). Structural comparisons by superposition of the AAT wild type and mutant molecular coordinates appear highlighted in the square insets (**a** to **f**). In **d**, reference (GlcNAc)2 glycan from (PDBid: 7API) is displayed in sticks. Residues (in sticks) are numbered according to the processed mature form of the protein

**Fig. 3:**
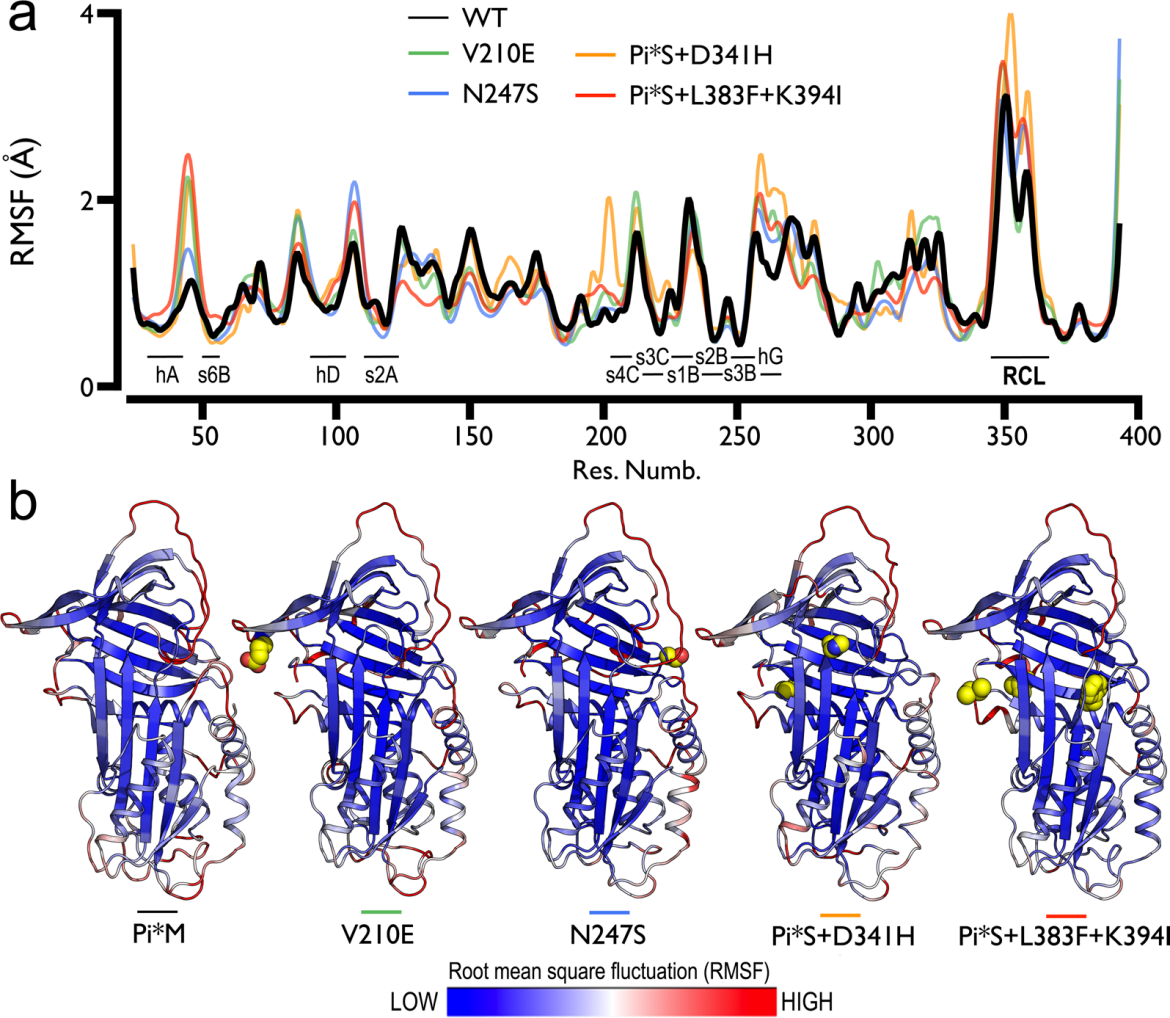
Molecular dynamics (MD) trajectory analysis of human AAT1 and identified mutants. **A** Root mean square fluctuation (RMSF) of Cα atom coordinates throughout 1 μs of MD for the wild-type (WT) human AAT and mutants V210E, N247S, Pi*S + Asp341His and Pi*S + Leu383Phe + Lys394Ile molecular models (color legend). **B** Cartoon representation of the WT and mutant’s average structures colored according to the fluctuation of atomic coordinates during MD production. Mutated residues at each monomer are displayed as yellow vdW spheres

Newly discovered AAT missense variants were localized both on the interaction surface and the hydrophobic core of the protein (Figs. 2 and 3). Distribution of mutations across the structure revealed Val210Glu at the solvent exposed s4C strand and close to the “gate” region. Asn247Ser was located on the accessible surface, which is important for glycan attachment. On the other hand, Asp341His, Leu383Phe and Lys394Ile were mapped close to the “breach” and “shutter” regions (Fig. 2).

The MD simulation method was applied for analyzing the structural effect of the mutational changes. The small deviation of the protein backbone atoms during 1 microsecond of simulation indicates that all amino acid replacements were tolerated, with an overall root mean square deviation < 2 Å in all molecular structures (see Table 3 and Additional file 1: Fig. S1). Most fluctuating regions correspond to the reactive center loop (RCL, residues 344–362 of the mature protein) and other non-structured regions in accordance with the weak electron density observed in the X-Ray structures [39] (see Fig. 3).

**Table 3:** Root mean square deviation (RMSD, in Å) of protein backbone atomic coordinate displacement during 1 μs MD simulations for the Wild-Type (WT) human AAT and mutants V210E, N247S, Pi*S + Asp341His and Pi*S + Leu383Phe + Lys394Ile water-solvated molecular systems. Average (Avg.), Standard deviation (Sd.) and minimum and maximum values (min. max.)

MD simulation structural models	Avg. RMSD_bkbn_ (Å)	Sd. (Å)	min. (Å)	max. (Å)
WT-AAT	1.838	0.175	0.562	2.275
V210E	1.850	0.217	0.536	2.382
N247S	1.754	0.178	0.396	2.260
Pi*S + Asp341His	1.996	0.258	0.477	2.733
Pi*S + Leu383Phe + Lys394Ile	1.936	0.297	1.141	2.909

**Fig. 4:**
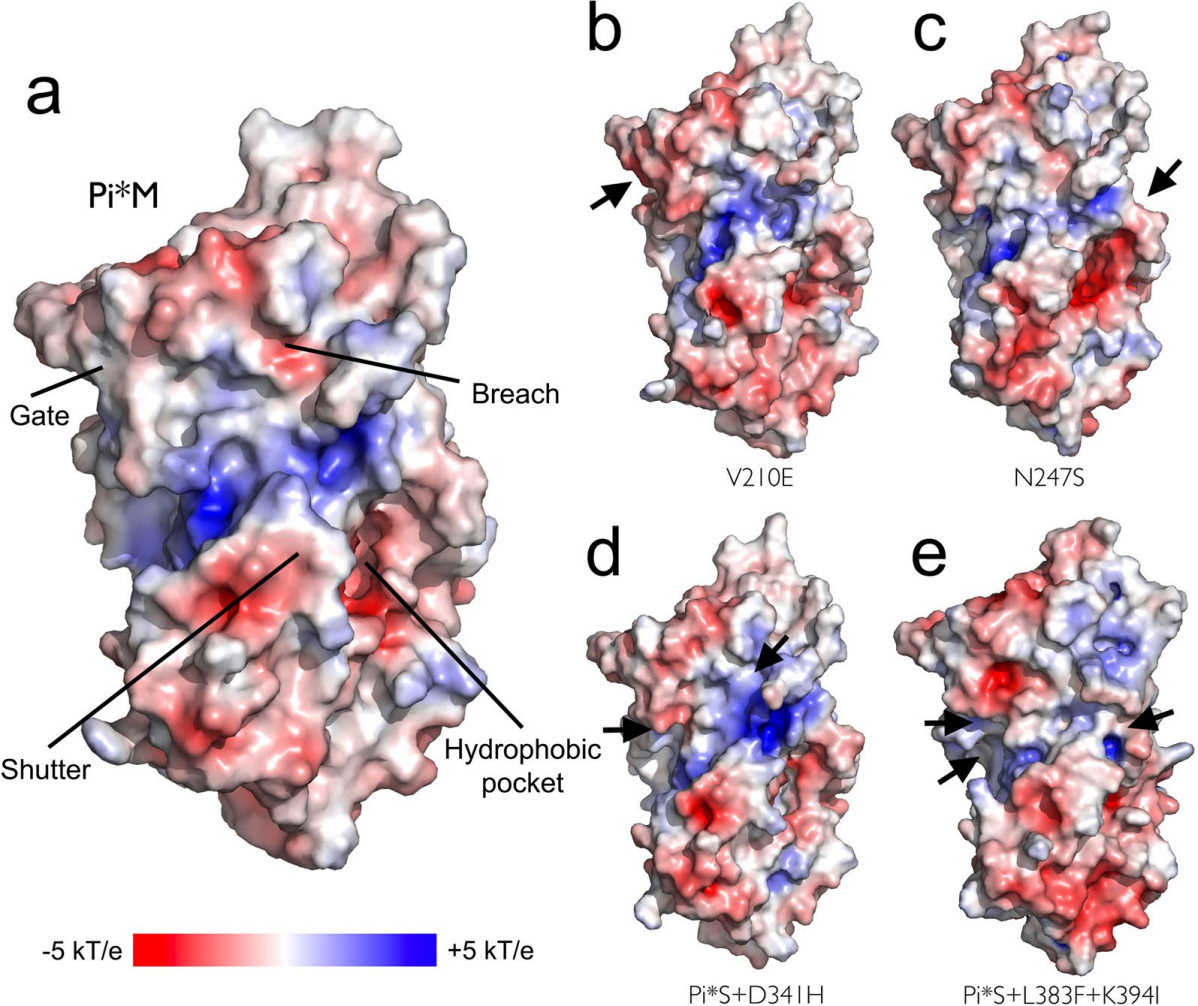
Surface representation of the wild-type (WT) human AAT and mutants V210E, N247S, Pi*S + Asp341His and Pi*S + Leu383Phe + Lys394Ile molecular structures (**a** and **e**). Molecular solvent accessible surfaces are colored by the electrostatic potential calculated using the APBS program with nonlinear Poisson-Boltzmann equation and contoured at ± 5 kT/e (negatively and positively charged surface areas in red and blue, respectively). Black arrows indicate the location of the substitutions in each mutant

MD analysis revealed the reshaping of local interactions around the investigated substitutions that have varying effects on AAT conformational flexibility, hydrophobic packing, and electronic surface properties (see Figs. 3 and 4). Substitution of the hydrophobic Val by the negatively charged Glu at position 210 promotes new electrostatic interactions of the s3C-s4C β-hairpin with the C-terminal (Fig. 2a). This change increases the negative charge on the “gate” region regarding the wild-type protein (Fig. 4a, b). On the other hand, the asparagine by serine mutation at the surface accessible N-glycosylation motif (Asn247-x-Thr) showed a moderate impact on the structural dynamics of the protein (Fig. 3). However, this position is crucial in the glycosylation profile of the protein and the loss of the glycan attachment site could affect the plasma half-life and stability of the protein (Fig. 2d) [40].

The most severe structural changes were observed in the double- and triple-mutant (Pi*S + Asp341His and Pi*S + Leu383Phe + Lys394Ile) molecular models. In both cases, the change of the negatively charged Glu by the hydrophobic Val at position 264 disrupted a salt bridge interaction with Lys387 and modified the relative orientation of helix hG (Figs. 2c and 3). In addition, the substitution of the negatively charged Asp341 by a positive His modified the molecular interactions and electrostatic landscape proximal to the “breach” region, which is essential for RCL insertion upon proteolytic cleavage (Figs. 2b, 4a, d).

Finally, the changes at position 383 reshaped the hydrophobic core and affect the electrostatic interactions towards the C-terminal region of the mutated protein. The Leu383 form part of a highly conserved sequence of 9 amino acids (residues 383 through 391) in the central core of the protein, chain s5B (Fig. 2f). As observed in the MD simulation experiments, the bulky aromatic Phe residue in this position induced interactions with other hydrophobic/aromatic residues (e.g., Leu103, Leu112, Phe190 and Phe384) modifying the hydrophobic pocket topology and the flexibility in the nearby hD-s2A region (Figs. 3, 4a, e). On the other hand, the functional role of Lys394 is uncertain due to the great flexibility observed in the MD experiments at the C-terminus residues (Figs. 2e, 3). This residue is not conserved among different species and its location and interactions are not preserved in time, suggesting a minor functional role [41].

## Discussion

The repertoire of AATD causing mutations has expanded over the last years, and to date, it is acknowledged that along with the major disease risk genotypes, ZZ and SZ, there are many others resulting from different combinations with rare deficient alleles. Indeed, previous studies have shown that rare mutations can be detected in up to 17% of clinically cases and, as for Z and S alleles, several low-frequency pathogenic variants have been segregating in human populations for hundreds or thousands of years [42, 43].

The emergence of new genomic technologies has boosted the identification of rare genetic variants with high accuracy and at a very reasonable cost, opening the way to the precision medicine healthcare approach. However, the major bottleneck for the effective use of this information in clinical practice lies in accurate interpretation of the functional consequences of the newly identified variants. Consequently, in this study we developed a method that combines biochemical, molecular biology and computational structural analysis for the interpretation of individuals carrying mutations on the *SERPINA1* gene.

We analysed a total of five mutations (four not previously described) in six subjects who presented moderate to severe AATD: Gly95Alafs*18, Val210Glu, Asn247Ser, Pi*S + Asp341His and Pi*S + Leu383Phe + Lys394Ile. All changes, except for the premature stop codon at position 95 of the mature protein, were studied at a structural level. The AAT missense mutations investigated were localized at interaction surfaces as well as at the hydrophobic core of the protein. Three variants, such as the Val210Glu, modify the electrostatic surface of the protein. Compared with Val, the Glu residue displays a negative charge, which reduces the extent of hydrophobic interactions by the s3C-s4C connecting turn close to the “gate” region. This observation agrees with other computational modelling studies that indicated an elevated pathogenic potential for this change [21].

A newly identified variant was also located on the surface of the protein. Human plasma AAT is normally fully glycosylated at three asparagine residues (46, 83, and 247) [6]. Thus, the Asn247Ser substitution interferes with the glycosylation signal, which is readily seen by IEF experiment, showing the observed band pattern to be altered. This change is distanced form the RCL and loss of the glycan groups has not been shown to alter the metastable state of AAT. However, it has been shown that some mutant variants which are glycosylated differently compared to normal AAT may be proteolytically degraded preferentially in the endoplasmic reticulum [44, 45]. Moreover, changes in glycan pattern could affect the half-life of the protein, aggregation kinetics and the immune modulatory properties of AAT [7]. The detection of CP was very low and similar to the concentrations observed in normal individuals PiMM.

Two other variants appeared in cis-configuration with the S variant and had a null-phenotype behaviour. The patient carrying the Pi*S + Asp341His presented severe AATD and he also presented the highest concentration of CP comparing with the other variants being similar to the concentration observed in patients PiSZ [19]. His genotype was a combination of three variants: PI*S in cis-configuration with the new mutation Asp341His (allele 1) and PI*Z (allele 2). This patient was a former smoker and had been diagnosed with bronchiectasis with mildly impaired lung function and recurrent pneumonia. According to AAT serum levels and the phenotype obtained, the combination of the S allele with Asp341His led to a new variant with features of null-phenotype behaviour. These results are in accordance with those obtained by Matamala et al*.* who described PI*S-plus alleles which modify the properties of the Pi*S allele in terms of higher intracellular retention [18]. In contrast to null alleles, which cause a complete abrogation of AAT production, these 'PI*S-plus' described by the authors, produced the AAT protein, but this protein is strongly retained intracellularly and for that reason is not detected in the serum of carriers. According to the MD analysis, Asp341His modify the structural packing and surface electrostatic properties of the protein around the “breach” region. Similar to what occurs in the Pi*S and Pi*Z variants, these changes would lead to aberrant conformations, disrupting the folding pathway of the protein [12]. The last variant causing a null-phenotype was the Pi*S + Leu383Phe + Lys394Ile. The carrier of these changes presented low AAT serum levels and low CP concentrations, similar to the concentrations observed in PIMM individuals. This patient also presented severe COPD, alcoholic liver disease and a clear MM IEF pattern, indicating that the combination of the S variant with the two novel mutations modified the secretion of the protein. Both changes were mapped on the C-terminal region, Lys394 being the last amino acid of the protein. In our computational experiments, this mutant modified the environment of the “hydrophobic pocket” and increased the dynamics of the RCL. Thus, changes in this preserved region may have significant effects on the secretion of the protein [46].

Owing to the considerable diversity of AAT-deficient variants, the present study aimed to provide a practical framework for the identification and analysis of new AAT mutations. This approach successfully combines the use of clinical data derived from standard laboratory tests, phenotyping, genotyping assays, and supporting computational modelling tools to delineate the impact of new SNV in the population. In this way, the determination of the functional impact of these rare deficient phenotypes will help to establish the requirements for the clinical management of serpinopathies. Additionally, the identification of these novel mutations should be incorporated in the diagnostic tools for the screening of AATD [47].

## Conclusions

Our results characterized five variants of the *SERPINA1* gene, four of them previously unkonwn, which define new alleles contributing to the deficiency of AAT. Rare variants might be more frequent than expected, and therefore, in discordant cases, standardized screening of the S and Z alleles’ should be complemented by gene sequencing and structural analysis. The use of conventional MD simulations, albeit useful to describe the changes on local interactions produced by mutations, is limited by the time-scale and impossibility to overcome large energy barriers. Thus, the application of enhancing sampling methods (e.g. metadynamics) should be considered for the study of the conformational dynamics of AAT in future developments. Nevertheless, we believe the computational analysis presented here, even with its limitations, provide valuable information that can be used in hypothesis generation of the pathogenicity of rare SNVs. All of the SNVs were previously recorded in the database of the National Center for Biotechnology Information of single nucleotide polymorphisms (dbSNP) and/or in the literature [19, 20, 22–26].

## Supplementary Information

**Additional file 1: Figure S1.** Stability of the AAT molecular dynamics trajectories. a. The root mean square deviation (RMSD) of backbone protein atoms during the 1 μs of MD simulations for the wild type (WT) and mutant structures (color legend). b. Radius of gyration (RG) of protein coordinates as a function of time for each simulated structure.

## Data Availability

Data are available from the authors under request.

## Abbreviations

AAT: Alpha-1 antitrypsin
AATD: Alpha-1 antitrypsin deficiency
ALAT: Alanine-aminotransferase alanine-aminotransferase
AST: Aspartate-aminotransferase
APBS: Adaptive Poisson-Boltzmann Solver
COPD: Chronic obstructive pulmonary disease
CP: Circulating polymers
CRP: C-reactive protein
dbSNP: Single Nucleotide Polymorphism Database
ELF: Enhanced liver fibrosis
FEV1: Forced expiratory volume in one second
FVC: Forced vital capacity
IEF: Isoelectric focusing
PI*: Protease inhibitor
GGT: Gamma-glutamyl transferase
MD: Molecular dynamics
MOE: Molecular Operating Environment
RCL: Reactive central loop
SPC: Simple-point charge
