# Supplementary figures and images for: New variants of alpha-1-antitrypsin: structural simulations and clinical expression
Source: Respir Res. 2022 Dec 10;23:339. doi: 10.1186/s12931-022-02271-8 (PMC9741788; doi:10.1186/s12931-022-02271-8)

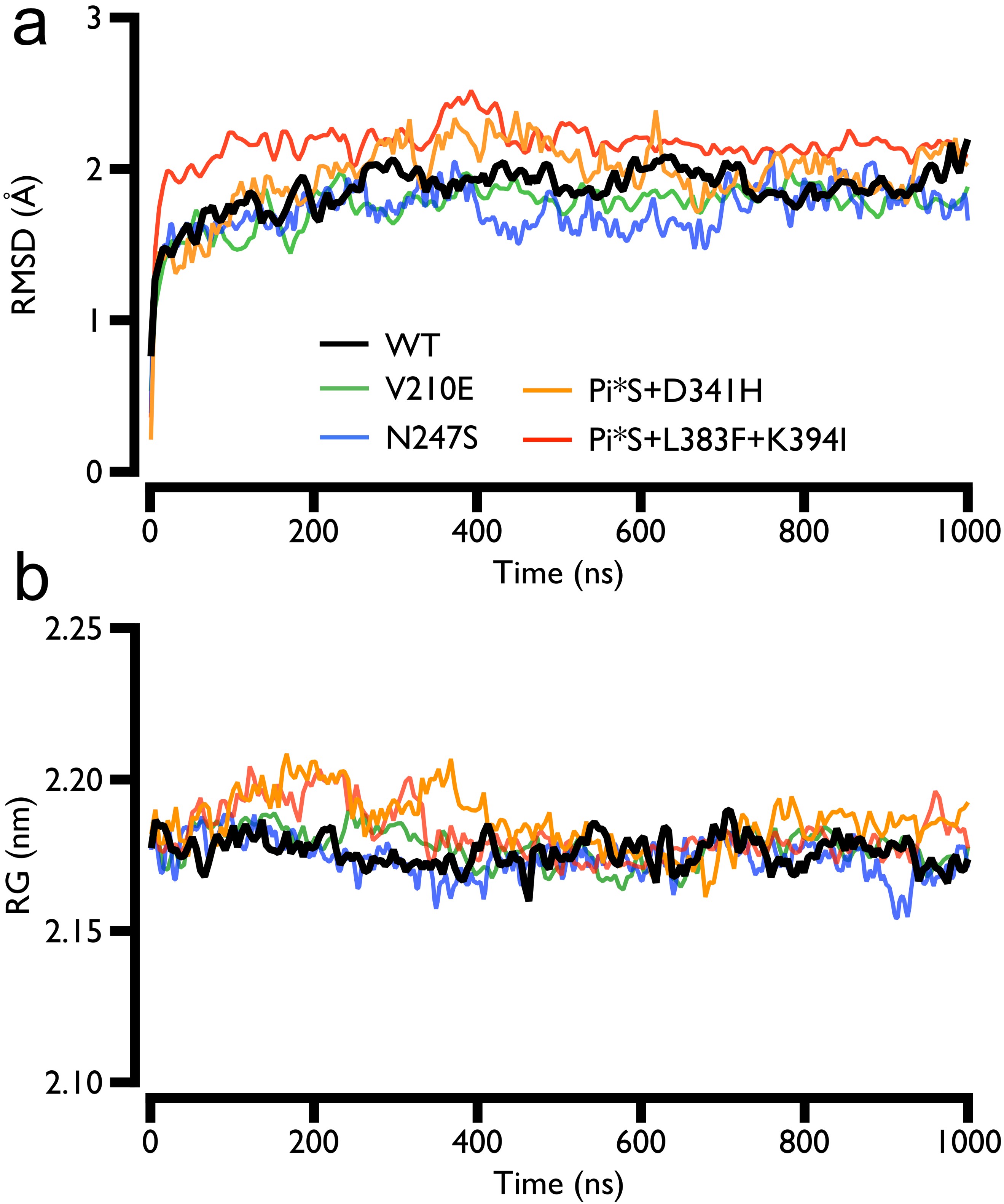

Supplement: Supplementary file 1 — Additional file 1: Figure S1. Stability of the AAT molecular dynamics trajectories. a. The root mean square deviation (RMSD) of backbone protein atoms during the 1 μs of MD simulations for the wild type (WT) and mutant structures (color legend). b. Radius of gyration (RG) of protein coordinates as a function of time for each simulated structure. [file 12931_2022_2271_MOESM1_ESM.jpg]
